# Convergence and divergence of genes informed by common and rare variants of autism spectrum disorders in tissue-specific pathways and gene networks

**DOI:** 10.1038/s41398-026-03824-x

**Published:** 2026-02-06

**Authors:** Cameron Gill, Yanning Zuo, Daniel Sung-min Ha, Russell Littman, Jason Hong, Jenny Cheng, Montgomery Blencowe, Susanna Sue-Ming Wang, Weizhe Hong, Ye Emily Wu, Xia Yang

**Affiliations:** 1 Department of Integrative Biology and Physiology, UCLA, Los Angeles, USA; 2 Neuroscience Interdepartmental Ph.D. Program, UCLA, Los Angeles, USA; 3 Department of Neurobiology, UCLA, Los Angeles, USA; 4 Department of Biological Chemistry, UCLA, Los Angeles, USA; 5 Department of Bioengineering, UCLA, Los Angeles, USA; 6 Brain Research Institute, UCLA, Los Angeles, USA

**Keywords:** Autism spectrum disorders, Neuroscience, Genomics

## Abstract

The genetic heterogeneity of autism spectrum disorder (ASD) presents significant challenges in understanding its pathogenic mechanisms, as the genetic risk involves numerous common variants and rare de novo or inherited variants. Prior research has mainly focused on identifying rare variants and their impact on neurodevelopment and neuronal functions in cortical brain regions. By contrast, common variants, which contribute substantially to ASD heritability, remain understudied, suggesting a need to consider both variant types to understand ASD’s genetic mechanisms. Previous studies have also implicated subcortical brain regions and peripheral digestive and immune systems, but tissue-specific mechanisms remain unclear. We address these knowledge gaps by identifying gene networks, pathways, and key regulators informed by ASD common variants in brain and peripheral tissues, further examining whether these networks also capture genes informed by rare variants. Our approach integrates genome wide association study (GWAS) summary statistics, tissue-level genetics of gene expression, and gene coexpression and transcriptional regulatory networks across ~50 tissues. Our multitissue, multiomics analysis reveals that key brain regions and networks crucial for synaptic signaling and neurodevelopment are enriched for both rare and common variants, whereas peripheral tissues, such as the digestive and immune systems, are primarily informed by common variants. This partitioning of key tissues and biological pathways into core (targeted by both variant types) and modifying components provide insight into ASD heterogeneity. We also identified central gene network regulators, such as *SYT1* and *ADD2*, which may orchestrate the effects of both common and rare ASD genetic risk factors on ASD pathogenesis.

## Introduction

Autism spectrum disorder (ASD) is a complex neurodevelopmental condition that manifests through social and communication deficits and various behavioral abnormalities [1, 2]. The prevalence of ASD has grown in recent years, likely due to improved diagnosis, with the latest epidemiological estimates suggesting more than 50 million people worldwide with some form of autism [3]. The current autism diagnosis criteria from the American Psychiatric Association’s *Diagnostic and Statistical Manual of Mental Disorders, Fifth Edition* include social-communication challenges (e.g., difficulties in social-emotional reciprocity, nonverbal communication, and relationships) and behavioral characteristics (e.g., repetitive movements, strict routines, and sensory sensitivities), and it categorizes ASD into three severity levels [4].

The wide range of social and communication deficits, behavioral abnormalities, and varying levels of severity indicate a diversity in ASD pathogenesis. This is likely rooted in substantial genetic heterogeneity as numerous rare mutations and common genetic variants with varying effect sizes have been identified in ASD [5]. Despite possessing a greater risk of causing significant autism syndromes, rare variants comprise of only ~1% of patients with autism [6]. Some studies suggest that the percentage of ASD heritability that can be explained by rare variants is up to ~5%, but estimates vary [7, 8]. Common variants in contrast, are more prevalent and have been estimated to collectively contribute to >50% of ASD heritability despite smaller effect sizes associated with individual variants [5, 9]. Using single nucleotide polymorphisms (SNPs) studied in genome-wide association studies (GWAS), the latest SNP-based estimate for heritability explained by common variants is ~12% [10]. Common variants can be summarized into an individual’s polygenic risk score to quantify the genetic predisposition to ASD [11].

Previous studies have identified key brain regions and their associated molecular and cellular pathways involved in ASD pathogenesis. The frontal and temporal cortical regions have shown abnormal gene expression patterns in autistic patients compared to typically developing individuals [12]. Additionally, two regions vital for complex cognitive processes, the anterior cingulate cortex and the amygdala, have exhibited decreased neuronal activity and abnormal growth, respectively, in autistic patients [13, 14]. There has also been evidence for non-symmetric development in the lateral ventricles and hippocampus [15]. Other implicated brain regions include the prefrontal, parietal, and visual cortices, the cerebellum, and the caudate nucleus [16, 17]. Interestingly, peripheral systems have also been implicated in ASD. For example, immune dysregulation has been associated with altered neurodevelopment and behavior [18]. The microbiota-gut-brain axis has also been explored given the interactions between commensal bacteria, immune cells, enteric nerves, and neurotransmitters, and the observation that ASD patients frequently present with gastrointestinal complications [19].

Despite these findings, the specific tissues, genes, and pathways underlying ASD are incompletely understood, and no effective treatments currently exist [20]. To address this, examining available omics data in a tissue-specific context is crucial as it may reveal relevant and potentially causal disease-associated mechanisms informed by common or rare variants. With this in mind, we hypothesize that ASD follows an omnigenic model of pathogenesis, where hub genes with large effect sizes interact with peripheral genes with smaller effect sizes via interconnected networks [21]. It is plausible that rare variants are enriched among the hub genes across essential tissues whereas common variants are enriched among peripheral genes within a broader range of tissues. Therefore, elucidating how common and rare variants of ASD converge and diverge in tissue-specific gene networks will identify key tissues and gene drivers within gene regulatory networks, providing further insights for future mechanistic and therapeutic studies. Additionally, as typical GWAS only focuses on the genome-wide significant loci with large effect sizes, common variants with moderate or subtle effects are missed. To test the omnigenic model, it is important to consider the full spectrum of GWAS summary statistics. We have established the foundation for this approach in multiple method papers and application studies in various disease areas [22–31].

In the current study, we integrate common variants from the full summary statistics of the largest GWAS of ASD to date [10], with tissue-specific gene regulation in the form of expression and splicing quantitative trait loci (eQTLs, sQTLs) and gene coexpression and regulatory networks to identify networks, pathways, and key regulators affected by common variants. We further compare these findings with genes informed by ASD rare variants to uncover commonalities and differences between the two types of variants in terms of tissue-, pathway-, and network specificity to distinguish between core and modifying mechanisms.

## Methods

### Analysis overview

**Fig. 1:**
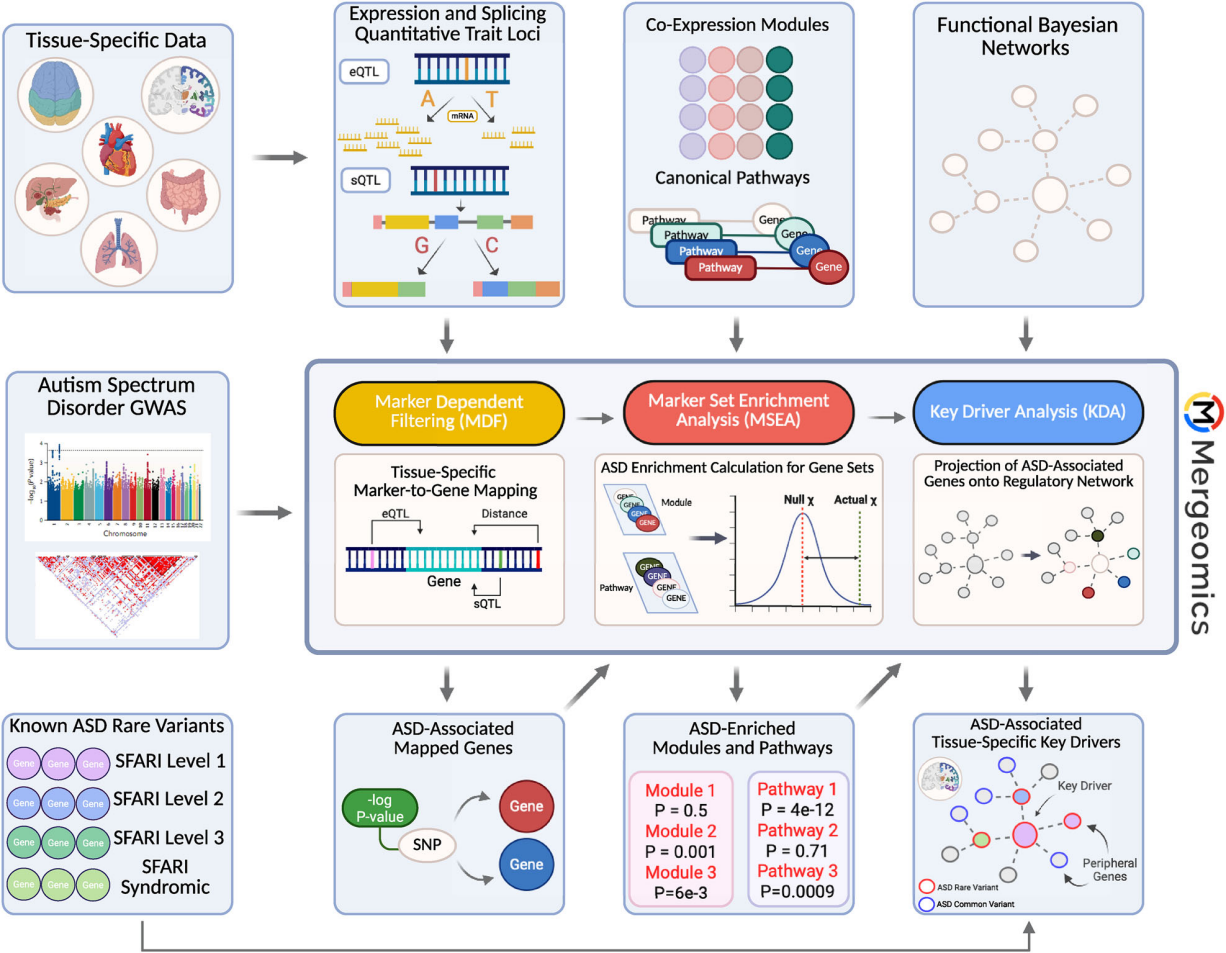
Analysis workflow. The first step of the overall analysis pipeline for Mergeomics is marker dependent filtering (MDF) to correct for linkage disequilibrium (LD) among GWAS SNPs. Summary statistics from the genome-wide association study are taken with tissue-specific eQTL and sQTL data to map genetic markers to corresponding genes in each tissue. ASD-enriched modules, or lists of genes that are found to have an association with ASD, are identified in marker set enrichment analysis (MSEA) by organizing the marker-mapped genes from MDF into coexpression gene sets and assessing their enrichment against a null distribution. By assessing their disease enrichment in a gene regulatory network, important regulatory genes are identified in key driver analysis (KDA). This figure was created with BioRender.com.

We utilize a multiomics integration tool, Mergeomics, for our analysis of ASD [32, 33], as its focus on integrating genetic variants with functional genomics to retrieve potential causal mechanisms is well suited for the goal of the study. The robustness of this tool has been substantiated by experimental validation of its computational predictions, and it has been successfully applied to the analysis of multiple complex diseases [34–37]. Briefly, we integrated full summary statistics of an ASD GWAS with tissue-specific expression and splicing quantitative trait loci (eQTLs/sQTLs) and tissue-specific gene coexpression networks to allow for the ranked identification of pathways and gene subnetworks most associated with ASD based on common variants examined in the GWAS (Fig. 1). The pipeline then performs a key driver analysis to determine network hub genes, termed “key drivers”, whose neighboring networks are enriched for disease-associated genes within interconnected gene regulatory networks. The outputs of Mergeomics include a ranking of biological pathways and subnetworks informed by ASD GWAS common variants in a tissue-specific manner, and a visualization of key drivers within tissue-specific disease subnetworks. Advantages of Mergeomics are: i) it utilizes the full GWAS summary statistics representing the spectrum of disease association strengths of genetic variants (strong, moderate, weak signals in the omnigenic model), and ii) it contains a unique test statistic that summarizes disease association enrichment at multiple quantile thresholds to derive stable statistics that are less dependent on any given GWAS significance cutoff to account for variability in sample size and statistical power. Figure 1 depicts our overall pipeline, datasets utilized, and the three steps of the Mergeomics analysis that are discussed in further detail below: marker dependency filtering (MDF), marker set enrichment analysis (MSEA), and key driver analysis (KDA).

### ASD common variant GWAS summary statistics

We used the full ASD GWAS summary statistics for over 9 million SNPs with association p-values, derived from a study that included >18,000 ASD cases and ~28,000 controls from a Central European (CEU) population [10]. Summary statistics from this study are publicly available and were downloaded from https://ipsych.dk/en/research/downloads/data-download-agreement-ipsych-pgc-asd-nov2017. In their study, quality control and GWAS analyses were performed using Ricopili, PLINK, and METAL pipelines, adjusting for batch effects and principal components to account for population stratification [38–40]. SNPs with minor allele frequencies (MAF) ≥0.01, imputation INFO scores ≥0.7, and effective sample size >70% were retained for analysis. Thus, our analysis only included common variants, as rare variants (MAF < 0.01) were excluded by definition. We chose to use this dataset because it is the largest ASD GWAS to date. However, we note that the population is primarily CEU and may have limited generalizability to other populations.

Importantly, we analyzed the full GWAS summary statistics from this GWAS study rather than solely the genome-wide significant loci from their analysis in order to better reflect the omnigenic model. Considering the combined contributions of strong, moderate, and weaker loci across gene networks is likely to capture a larger proportion of ASD heritability compared to genome-wide significant loci.

### Marker dependency filtering (MDF)

SNP-to-gene mapping was performed using ASD GWAS SNPs and tissue-specific expression and splicing quantitative trait loci (eQTLs and sQTLs) from the Genotype Tissue Expression (GTEx) project database [41], and distance-based mapping (±20 kb) was used as an alternative mapping method to capture cis-regulatory relations. The complete list of tissue-specific eQTL/sQTL data for 49 tissues used as input for the Mergeomics analysis are in Supplementary Table 1. We corrected for linkage disequilibrium (LD) to filter known dependencies between SNPs based on an LD cutoff threshold of r^2^ > 0.5 (the default threshold for Mergeomics based on performance benchmarking) from the CEU population, as the ASD GWAS population is mainly CEU. The output of MDF contained tissue-specific SNP-to-gene mappings based on eQTL, sQTL, and distance-based methods, with their ASD association strengths represented by *-log10* p-values after filtering out SNPs in LD by keeping the SNP with the strongest GWAS association within each LD block. The LD clumping step was done to remove redundant SNPs entering downstream analysis, and the mapping of SNPs to genes via eQTLs and sQTLs was performed to offer tissue and gene annotations for GWAS SNPs based on data-driven potential tissue-specific gene regulation. No GWAS p-value cutoffs were applied, ensuring a comprehensive range of association signals for downstream null distribution estimation in MSEA.

### Tissue-specific weighted gene coexpression network analysis (WGCNA) to define data-driven functional gene sets in each tissue

To group genes with functional relevance in individual tissues in a data-driven manner, we used the transcriptome data from the GTEx database to construct tissue-specific WGCNA gene coexpression modules [42]. Typically, these modules contain genes that are coexpressed and functionally related. This provided a means of placing our ASD-associated GWAS genes mapped from MDF into categories that have biological relevance in individual tissue contexts. These modules were functionally annotated through pathway enrichment analysis using KEGG [43], Reactome [44], and BioCarta [45] databases. We constructed WGCNA networks for all tissues in GTEx. To facilitate the interpretation of tissues that generated results after marker set enrichment analysis, we categorized the tissues into seven systems: Adipose/Immune (subcutaneous adipose, visceral adipose, EBV transformed lymphocytes, spleen, whole blood), Brain (amygdala, anterior cingulate cortex, caudate, cerebellar hemisphere, cortex, frontal cortex, hippocampus, hypothalamus, nucleus accumbens, putamen, pituitary, spinal cord, substantia nigra), Cardiovascular (aortic artery, atrial appendage, coronary artery, left ventricle, tibial artery), Digestive (esophagus mucosa, esophagus muscularis, gastroesophageal junction, liver, minor salivary gland, pancreas, sigmoid colon, small intestine terminal ileum, stomach, transverse colon), Endocrine (adrenal, ovary, pituitary, testis, thyroid), Female Reproductive (breast mammary tissue, endocervix, ectocervix, fallopian tube, ovary, uterus, vagina), and Male Reproductive (prostate, testis).

### Marker set enrichment analysis (MSEA)

We next used MSEA to assess whether tissue-specific WGCNA coexpression modules from GTEx tissues are enriched for SNPs showing stronger ASD GWAS associations compared to a null distribution derived from random gene sets. GWAS-mapped genes from MDF and tissue-specific WGCNA coexpression modules were used as input, with a chi-like statistic used as the enrichment test statistic to determine coexpression modules enriched in the ASD GWAS:

$${\rm{\chi }}=\mathop{\sum }\limits_{\mathrm{i}=1}^{\mathrm{n}}\frac{{\mathrm{O}}_{\mathrm{i}}-{\mathrm{E}}_{\mathrm{i}}}{\sqrt{{\mathrm{E}}_{\mathrm{i}}}+{\rm{\kappa }}}$$

Briefly, *n* represents the number of quantile points used to divide the GWAS SNPs into significant and non-significant groups. Rank-based quantiles were used instead of specific p-value cutoffs to mitigate the sample size influence on p-values. We used 10 quantiles ranging from 0.5 to an upper limit (*q*_upper_) adjusted by the median module size (*μ*). This upper limit was calculated as *q*_upper_ $=1.0-1.0/\mu$, allowing the quantile distribution to account for module size.

*O*_*i*_ and *E*_*i*_ represent observed and expected positive associations at each quantile point, with the difference divided by the square root of the expected count plus a stability parameter, $\kappa =1$, to account for extremely low expected counts. Thus, the calculated $\chi$ value is a sum of the output at each quantile point from a given test gene set or module. A null distribution was created by generating random gene sets matching the gene number of each coexpression module and calculating its $\chi$ value, with the following null hypothesis: *Given all distinct markers from a set of N genes, these markers contain an equal proportion of positive associations when compared to all the distinct markers from a set of N random genes* [32]. Z-scores were then calculated to measure the deviation of each module’s $\chi$ value from the null distribution’s mean, and enrichment scores were determined from these Z-scores to rank tissue-specific modules by their enrichment for ASD GWAS signals. P-values were calculated from the Z scores using a Gaussian distribution, and a false discovery rate (FDR) was estimated using the Benjamini-Hochberg method.

### Tissue-specific Bayesian gene regulatory networks to define gene regulatory relations

WGCNA coexpression modules focus on correlation but not regulatory information between genes. Therefore, to elucidate directional gene regulatory relations, we also constructed Bayesian networks from tissue-specific GTEx databases using the RIMBANet package [46] for all tissues, which incorporates priors from eQTLs, transcription factors to target relations, and mutual information to model gene-gene regulation. As Bayesian networks from individual datasets are typically sparse, networks from similar tissues were subsequently merged to derive composite networks for brain, digestive, cardiovascular, endocrine, immune, adipose, and reproductive tissues to reduce sparsity and ensure each network contains at least 10,000 genes. We further merged the immune and adipose tissue networks given the role of adipose tissue in immune regulation [47]. The merged networks and their corresponding constituent tissues and network sizes are shown in Supplementary Table 2.

### Key driver analysis (KDA)

We used KDA to identify key drivers (i.e., hub network genes) of ASD-associated gene coexpression modules from MSEA and their associated neighbors within tissue-specific Bayesian gene regulatory networks constructed from GTEx tissues. Key drivers were predicted by identifying hub nodes in the top 25% of edge connections. For each hub node and its subnetwork, KDA assessed enrichment in ASD-associated gene sets using a calculation similar to MSEA. The proportion of subnetwork nodes overlapping with ASD-associated modules was compared to a null distribution generated from reshuffled subnetworks of the same size to calculate an enrichment statistic. The outputted networks were ranked by their enrichment for ASD GWAS-informed gene sets from MSEA. Using EnrichR, a gene set enrichment annotation tool [48–50], we performed pathway enrichment analyses to understand the functions of predicted key driver subnetworks.

### Assessment of rare variants in tissue-specific key driver (KD) networks of ASD common variants

Genes informed by ASD rare variants were obtained from the Simons Foundation Autism Research Initiative (SFARI), an authoritative database in the ASD field which stratifies genes into four confidence levels: Level 1 (high confidence in their implication in ASD; presence of at least three de novo likely-gene-disrupting mutations at genome-wide significance or at least at FDR < 0.1), Level 2 (strong candidate for ASD association; genes with two reported de novo likely-gene-disrupting mutations), Level 3 (moderate evidence based on previous research; genes with a single de novo likely-gene-disrupting mutation), and Level Syndromic (genes with mutations associated with both ASD and a specific syndrome beyond the characteristics of ASD) [51]. We utilized the SFARI database as the resource for ASD rare variant genes because it incorporates findings across many large-scale sequencing studies, including the Simons Simplex Collection and databases such as AutDB [52, 53]. As a result, the database encompasses de novo mutations, exome and genome sequencing, and rare variant and CNV studies, which makes SFARI more inclusive than any individual rare variant study. Thus, it is well-suited for network-based analyses such as ours. The link to download the most updated dataset of genes and their associated variants and statistics can be found here: https://gene.sfari.org/database/human-gene/.

Within each tissue-specific KD subnetwork identified for ASD common variants, genes in the subnetworks that contain known rare variants were annotated based on information from the SFARI database. We considered the rare variants both as a collective across all four ASD levels and at each individual stratification level (i.e., Level 1, 2, 3, syndromic) for the analysis. Enrichment for rare variant genes within each common variant-informed gene subnetworks was assessed using Fisher’s exact test.

### KD subnetwork visualization

Cytoscape (version 3.10.1) [54] was used to visualize each of the top ranked KDs and their subnetworks. Genes mapped to common and rare ASD variants were annotated for each KD subnetwork.

### Heritability estimation of network contributions of brain and peripheral ASD networks

We applied the Heritability Estimation from Summary Statistics (HESS) method (v0.5) [55] to evaluate the contribution of the brain and peripheral networks associated with ASD from our network analysis to the SNP-heritability of ASD using the GWAS summary statistics from Grove et al. HESS estimates total and local SNP-heritability using LD information from reference panels and GWAS summary data. For each subnetwork (all significant modules from peripheral tissues or all significant modules across brain regions), we first performed step 1 of HESS to compute local heritability across approximately independent LD blocks. In step 2, we aggregated local estimates to obtain global SNP-heritability for each subnetwork. We used pre-computed LD matrices from European ancestry samples (1000 Genomes Project) and ASD GWAS summary statistics. Genomic control inflation (λGC = 1) was applied to correct for potential test statistic inflation.

## Results

### Marker set enrichment analysis (MSEA) reveals tissue-specific coexpression modules enriched for ASD GWAS signals

**Fig. 2:**
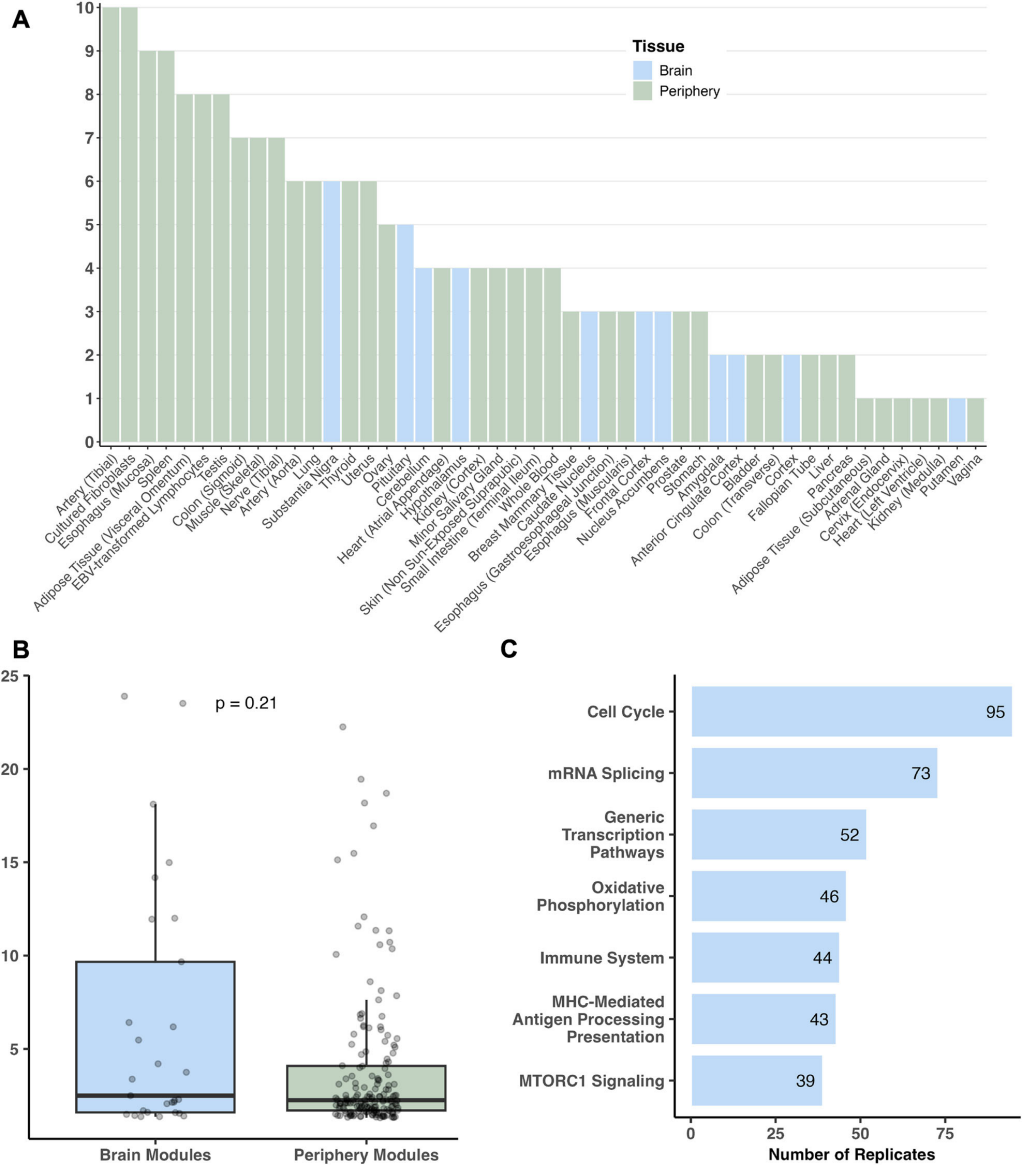
Informative tissues and pathways based on module enrichment for ASD GWAS signals. **A** Tissues were ranked by the number of WGCNA modules with FDR < 5%. for ASD GWAS association enrichment based on Marker Set Enrichment Analysis (MSEA). **B** No significant difference was observed in the ASD GWAS enrichment between brain and periphery coexpression modules in MSEA. The difference in the *-log10* FDR between brain and peripheral modules was calculated utilizing a two-sided Wilcoxon test given the non-parametric distribution of the data based on Shapiro-Wilk test (p-value < 2.2e-16). **C** The most consistent functional terms across significant coexpression modules from MSEA show cell cycle, mRNA splicing, immune system, and mTOR signaling pathways.

The MSEA analysis identified 48 tissues (11 brain regions, 37 peripheral tissues) out of all tissues analyzed from which at least one coexpression module was significantly enriched for ASD associations in GWAS at FDR < 5% (Fig. 2A). Across these tissues, there were 196 ASD-enriched WGCNA modules. Supplementary Table 3 shows the top 10 modules based on the statistical significance of ASD GWAS enrichment, where coexpression modules with diverse functional annotations from 5 brain regions and 5 peripheral tissues were observed. The complete list of modules are seen in Supplementary Table 4. The anterior cingulate cortex, which is involved in emotional regulation and cognitive control, and the amygdala, another region crucial for emotional response, contained two coexpression modules with the highest ASD enrichment. The top functional annotations for these two modules included protein activity, immune system, and neuroplasticity. The other top brain tissue coexpression modules were from the frontal cortex (Brodmann Area 9, [BA9]), cerebellum, and cortex, and were enriched for pathways involved in neuronal signal transduction and immune regulation. Together these findings support how impaired neuronal and immune functions play a significant role in ASD pathogenesis. Interestingly, peripheral tissues from the digestive system (liver, minor salivary gland), reproductive system (uterus, testis), and immune system (lymphocytes) contained highly significant coexpression modules relevant to mRNA splicing, immune pathways, cell cycle, and mammalian target of rapamycin (mTOR) signaling, which have been implicated in ASD [18, 56, 57]. When further examining the genes in these pathways with the strongest GWAS association p-values, we note an abundance of non-coding RNAs (ncRNAs) in addition to genes important for neuronal and immune functions. These results align with recent studies that implicate the roles of ncRNAs in ASD and other related disorders [58–62].

We next examined all significant modules across brain and peripheral tissues, expecting significant modules from brain tissues to collectively show higher levels of significance for ASD GWAS enrichment compared to modules from peripheral tissues given that ASD is a neurodevelopmental disorder. Surprisingly, we did not find significant difference in the average FDRs between brain and peripheral tissue modules (Fig. 2B), suggesting that peripheral tissues are not less informative than brain tissues in capturing ASD common variants in coherent functional modules. Across all significant modules, pathway annotation revealed a broad range of consistent pathways including cell cycle, gene regulation (particularly splicing), oxidative phosphorylation, immune system, and mTOR signaling (Fig. 2C).

**Fig. 3:**
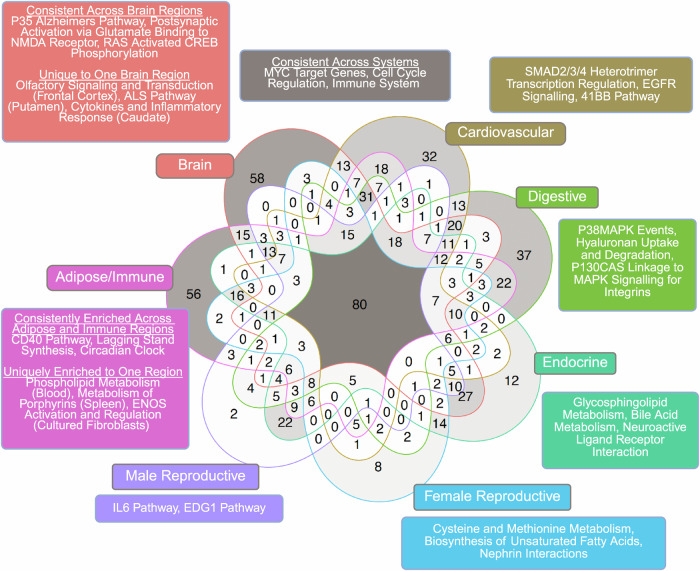
Shared and unique pathway terms overrepresented in significant coexpression modules in MSEA between 7 organ systems. The seven organ systems were Adipose/Immune, Brain, Cardiovascular, Digestive, Endocrine, Female Reproductive, and Male Reproductive. The pathways were ranked based on the median values of the *-log10* transformed adjusted p-values of the pathway derived from multiple coexpression modules, and the top three terms (where applicable) were subsequently depicted in the figure. Pathways shared across all organ systems or with Adipose/Immune and Brain systems were extensive enough that we categorized pathways based on whether they were shared across >3 tissues in a given organ system or >30 tissues across all organ systems, or if they were enriched only in one tissue within an organ system. This figure was created using ggVennDiagram and stylized with BioRender.com.

To further explore the pathways within the enriched coexpression modules of tissues with the most relevance to ASD, we categorized tissues into seven systems: Adipose/Immune, Brain, Cardiovascular, Digestive, Endocrine, Female Reproductive, and Male Reproductive. There were nearly 800 pathways across all tissue systems, among which 80 were shared across all seven systems (Fig. 3), mainly encompassing immune pathways, cell cycle regulation, cellular signaling, growth and proliferation, and protein interactions. Among the system-specific pathways, we observed crucial neuronal and signaling processes (postsynaptic activation via glutamate binding to NMDA receptor, Ras activated CREB phosphorylation, olfactory signaling and transduction) and neurodegenerative conditions (Alzheimer’s disease, Amyotrophic Lateral Sclerosis) for the brain; immune regulation involving the CD40 pathway and circadian clock for the Adipose/Immune system; glycosphingolipid metabolism and neuroactive ligand-receptor interaction for the Endocrine system; and hyaluronan uptake/degradation and MAPK signaling for integrins within the digestive system. The tissue-specific modules and processes suggest diverse and unique contributions of various systems to ASD pathogenesis.

We note that there was no significant overlap between the tissue-specific modules identified in MSEA and tissue-matched differentially expressed genes (DEGs) from previous transcriptomic studies [63–72] (Supplementary Table 5). This observation is consistent with previous studies of other complex diseases [27, 73], which also found that network modules but not DEGs were enriched for genetic variants. It is likely that DEGs better reflect changes downstream of disease, whereas coexpression modules capture causal disease mechanisms.

### Key driver analysis (KDA) identifies distinctions between brain and peripheral tissue associations in ASD

**Fig. 4:**
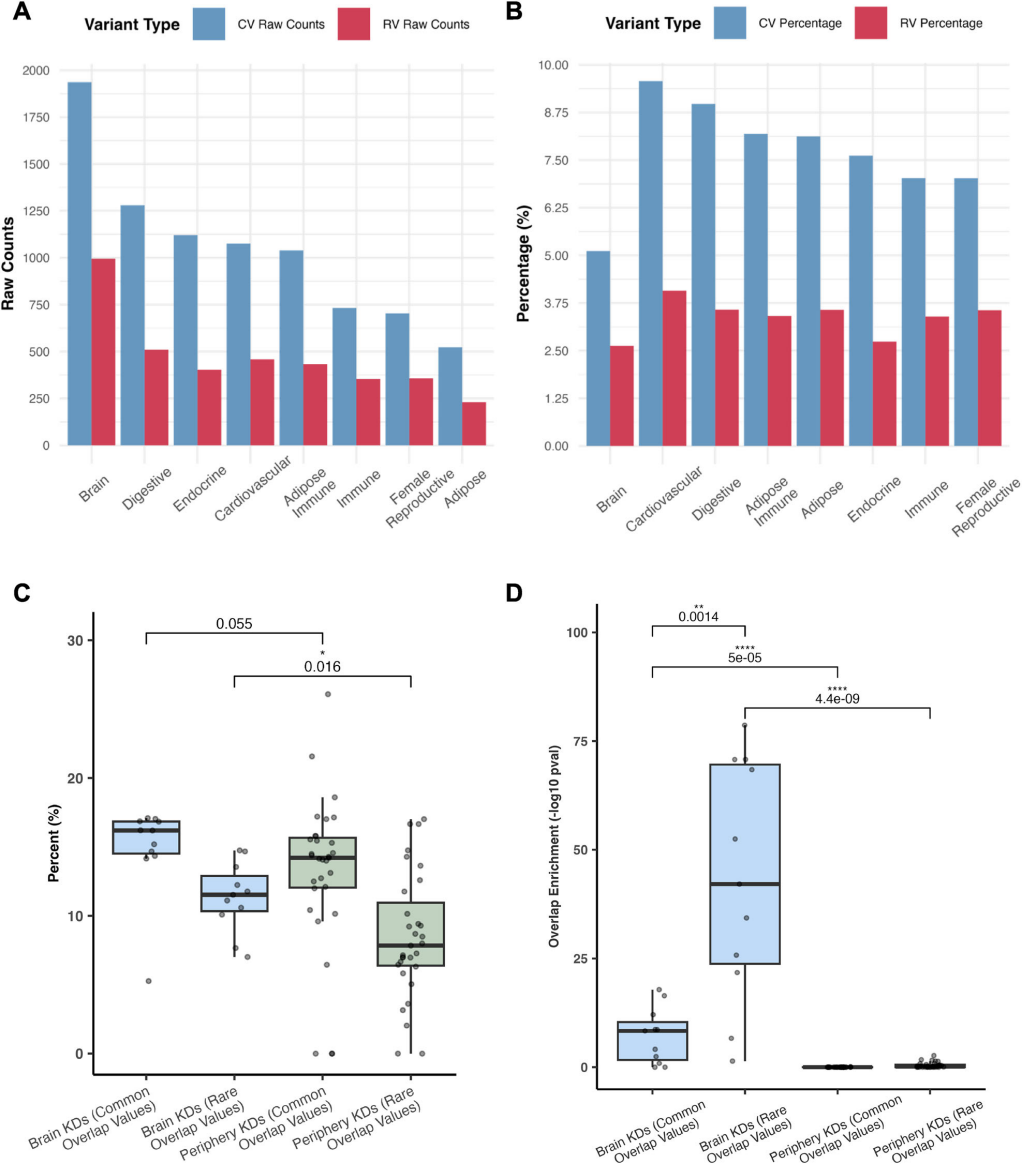
Comparison of brain and peripheral tissue networks and key drivers in terms of overlap with common and rare ASD variants. **A** Raw counts of the number of genes in each tissue network that were either common variants from the ASD GWAS (blue bars) or known rare variants from the SFARI database (red bars). **B** Normalized counts of the number of common variants (blue bars) or known rare variants (red bars) over total gene numbers in each network. **C** Comparison of percentages of key drivers overlapping with known ASD rare variants and common variants between brain and peripheral tissues. Each data point corresponds to the percentage of key drivers containing either rare or common variants from a given brain or peripheral tissue. Given the presence of outliers and the non-normal distribution of the data based on the Shapiro-Wilk test (p-value = 0.008316), we performed a one-sided Wilcoxon test to determine the significance between groups with the expectation of higher percentage values for KDs in the brain tissues than those of the peripheral tissues. **D** Comparison of rare or common variant enrichment scores among key drivers between brain and peripheral tissues. For each brain or peripheral tissue, the key drivers identified were first assessed for enrichment for rare variants and common variants of ASD using the hypergeometric test. Subsequently, the statistical significance of the rare/common variant enrichment from the hypergeometric test for all brain tissues were compared with that of all peripheral tissues using a two-sided Wilcoxon test due to the non-parametric nature of the data based on the Shapiro-Wilk test (p-value = 6.19e-16). CV Common Variant, RV Rare Variant, KD Key Driver.

Using the significant tissue-specific coexpression modules identified from MSEA, we performed KDA to identify tissue-specific key drivers (KDs). We also assessed whether the key drivers and their subnetworks capture both common and rare variants of ASD by intersecting the subnetworks with genes containing these variants. As seen in Fig. 4A, we observed higher numbers of rare and common variants in the key drivers from the brain Bayesian networks compared to the peripheral networks, supporting the importance of the brain in ASD, as expected. Besides brain KDs, digestive system KDs contain the second highest numbers of rare and common variants. However, when normalizing the gene counts of rare and common variants against the total number of genes in the peripheral and brain Bayesian networks, the normalized count was higher for peripheral tissue networks (Fig. 4B), which is likely due to the much smaller sizes of the peripheral tissue networks compared to more complex brain networks (Supplementary Table 2). This finding supports that peripheral tissue genes possess sizable contributions to the overall genetic burden of ASD.

We next focused on the identified KDs and sought to assess the differences in the degrees of statistical enrichment of common and rare variants among KDs between the brain and peripheral tissues. Figure 4C shows the percentage of key drivers across both brain and peripheral tissues that are also either known genes harboring rare variants from the SFARI database, or contain common variants from the GWAS. We observed a higher percentage of known rare ASD variants among brain tissue-derived KDs compared to peripheral tissue-derived ones. As the rare variants mostly affect brain development and neuronal functions and have larger effect sizes, our results align with the central role of brain tissues in the pathogenesis of ASD particularly for ASD driven by rare variants. By contrast, there was a similar percentage of common variants among KDs between brain and peripheral tissues, suggesting that common variants of ASD are less discriminative between the brain and the peripheral tissues.

We then statistically assessed whether the KDs were enriched for common and rare variants of ASD using the hypergeometric test. As shown in Fig. 4D, brain tissue KDs showed much stronger enrichment for both rare and common ASD variants compared to peripheral KDs, further supporting the importance of the brain in ASD. Moreover, the brain KDs showed a significantly higher enrichment for rare variants than for common variants. As rare variants have larger effect sizes than common variants, the observation of stronger enrichment of rare variants among brain KDs also supports the stronger influence of brain networks in ASD. When separating the ASD rare variants by level, we found that brain key drivers were significantly enriched for genes from each level, with Level 1 and 2 genes showing stronger statistical significance than Level 3 and Syndromic genes, and Level 1 genes displaying the highest fold enrichment (Supplementary Table 6). This pattern suggests that genes with stronger rare evidence for ASD association are more likely to be predicted regulators of ASD brain networks.

### Prioritization of KDs based on rare/common variant enrichment in KD subnetworks

**Fig. 5:**
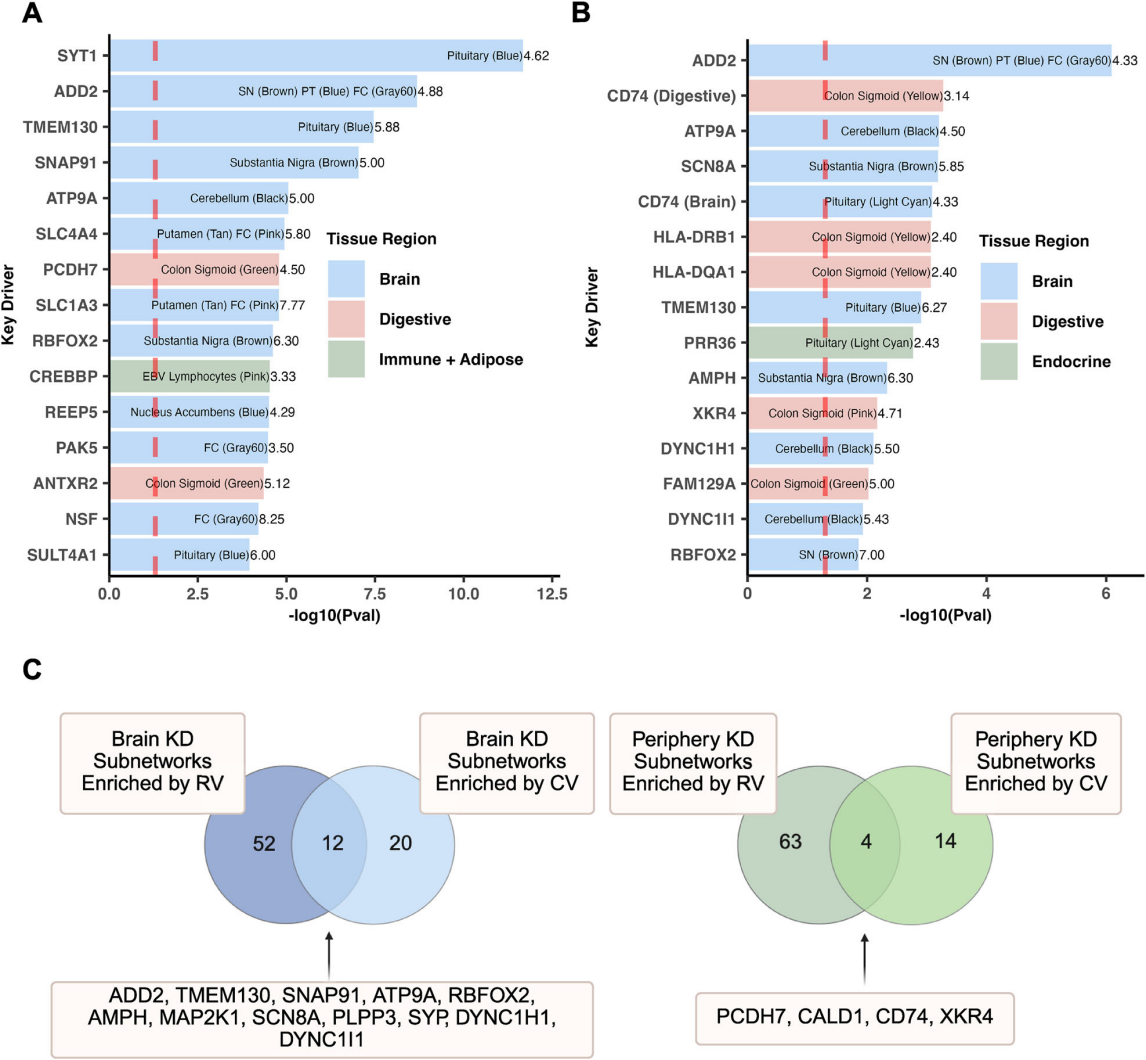
Key driver subnetwork characteristics: Variant enrichment and convergence across brain and peripheral tissues. **A** Top 15 key driver subnetworks ranked by rare variant enrichment. Key drivers were ranked by their *-log10* transformed p-value generated from hypergeometric testing of the key driver subnetwork and its overlap with SFARI rare variants. Within each bar is the key driver’s tissue(s) with the name of the ASD-associated coexpression module in parentheses. The numbers to the right of each bar display the fold enrichment for each subnetwork, which indicates the subnetwork’s enrichment for known rare variants. The background set of genes for this analysis were all genes in the GTEx transcriptome data for each tissue. **B** The same analysis as in (**A**), but ranking subnetworks by enrichment for ASD GWAS common variants. Fold enrichment values reflect overlap with common rather than rare variants. **C** Venn diagrams comparing key drivers enriched for rare variants and common variants in brain (left) and peripheral tissues (right). Key drivers with enrichment for both rare and common variants are listed for each tissue category. This figure was created with BioRender.com. SN Substantia nigra, PT Pituitary, FC Frontal Cortex, KD Key Driver, CV Common Variant, RV Rare Variant.

We prioritized KDs based on the significance of their subnetworks for common and rare variant enrichment. Among the top 15 KD subnetworks in terms of their rare (Fig. 5A) and common (Fig. 5B) variant enrichment, respectively, there was a higher abundance of brain tissue KD subnetworks (12 out of 15) that were enriched for rare variants, and an increasing representation of digestive tissue KD subnetworks (5 out of 15) that had enrichment for common variants. There was also a higher proportion of brain tissue KD subnetworks enriched for both rare and common variants (Fig. 5C; Supplementary Table 7), supporting convergence between rare and common variants in these KD subnetworks.

**Fig. 6:**
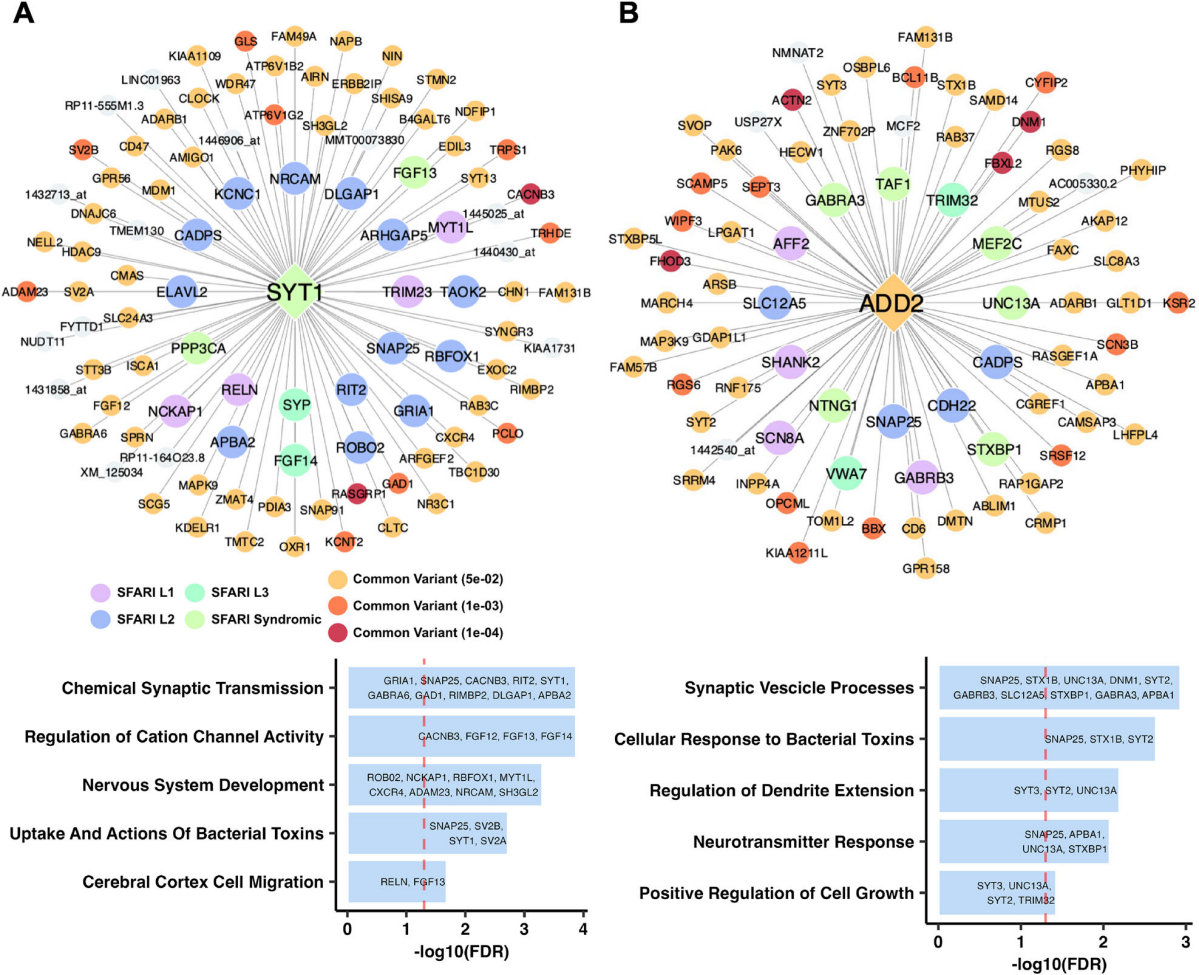
Brain tissue key driver subnetworks and pathway enrichment annotation terms. **A** Key driver subnetwork for Synaptotagmin 1 (*SYT1*), a key driver with high rare variant enrichment, and the associated pathways. **B** Key driver subnetwork for Adducin 2 (*ADD2*), a key driver with high rare and common variant enrichment, and the associated pathways. In (**A**, **B**), the key driver and its first-degree network neighbors are visualized using Cytoscape and colored based on their SFARI database ASD confidence level stratification or by their ASD GWAS common variant disease association strength. Uncolored nodes were genes within the respective Bayesian network but were not found to be associated with either grouping. EnrichR was used to analyze the key driver subnetwork genes to generate pathway annotation terms, with the top annotations displayed below each network. Terms were ranked based on their *-log10* false discovery rate.

Two brain tissue KD subnetworks stood out due to their high enrichment of either common or rare variants. Synaptotagmin 1 (*SYT1*, Fig. 6A) held the highest rare variant enrichment in its subnetwork across all KDs. Our key driver analysis corroborates its role in ASD as a known syndromic rare variant, and its subnetwork also contains both rare (e.g., *RELN, NCKAP1*) and common (e.g., *CACNB3, SV2B*) variants, suggesting a regulatory role in both variant types. *SYT1* is a synaptic vesicle membrane protein involved in calcium-mediated neurotransmitter release, thus playing a vital role in neuronal communication [74, 75]. It is a known syndromic ASD rare variant causing severe neurodevelopmental abnormalities. The top annotation terms from the *SYT1* subnetwork include synaptic transmission, cation channel activity regulation, and nervous system development, agreeing with this gene’s importance in neurodevelopment (Fig. 6A).

The subnetworks of several other top brain KDs, *ADD2* (Fig. 6B), *SCN8A, AMPH, ATP9A* (Supplementary fig. 1A-C), demonstrated enrichment of both variant types, thereby emphasizing the convergence between these variants orchestrated by these brain KDs. For instance, the ADD2 subnetwork had both rare (e.g., *SHANK2, SCN8A*) and common variants (e.g., *ACTN2, DMN1*) in its neighborhood (Fig. 6B). Adducin genes encode cytoskeleton proteins that are critical for osmotic rigidity and cell shape by regulating the formation of the spectrin-actin membrane skeleton [76]. *ADD2* is expressed in the brain, and its knock-out results in the loss of activity-dependent connection formation between neurons [77]. The top functional annotations of the ADD2 subnetwork include synaptic vesicle processes and dendrite extension regulation, which are key components of neurodevelopment (Fig. 6B). The function of *ADD2* itself and the functional annotations of its subnetwork support its relevance to learning and development and its potential contribution to ASD. *SCN8A*, a sodium voltage-gated channel gene essential for neuronal excitability, has been associated with neurodevelopmental disorders including ASD [78]. *AMPH*, a gene involved in synaptic vesicle cycling, is crucial for neurotransmitter release and thus may influence neuronal communication [79]. *ATP9A*, a phospholipid transporter involved in membrane trafficking and neuronal homeostasis [80, 81], is also a key candidate for further investigation of ASD pathogenesis.

In addition to the top brain KDs discussed above, our analysis also revealed a top KD within the endocrine Bayesian network, *PRR36*, which showed strong enrichment for common variants (e.g., *DNM1*, *MYT1*, *MAPK15*) but not for rare variants (Supplementary fig. 2A). While its precise role remains understudied, *PRR36*, or proline rich 36, has been associated with ASD-related genes such as *SHANK3*, which is implicated in synaptic organization and neurodevelopment [82]. Another top KD, *PCDH7* (encoding protocadherin; Supplementary fig. 2B), was identified from digestive tissue networks and its subnetwork is enriched for both rare (SFARI Level 1 gene *SETBP1*; Level 2 genes *CHRM3, KCMNA1, GPC4, STK39, PLN*; Level 3 gene *PDE3B* etc.) and common variants (e.g., *MITF, PDE3A, TSPAN2, THRB* etc.). *PCDH7* is important for cell adhesion, cell-cell interaction and signaling, and brain plasticity and synapse maturation [83, 84]. Its identification as a key driver in the ASD-enriched sigmoid colon module, along with its association with rare ASD variants and its enrichment for cell signaling pathways, suggests shared functions between brain and digestive tissues, with potential contributions to ASD via either or both systems.

### Contribution of brain and periphery ASD-associated networks to genetic heritability

We used Heritability Estimation from Summary Statistics (HESS) to estimate the contribution of SNPs within the peripheral and brain subnetworks using genes in all significant modules from each compartment to ASD heritability. The peripheral subnetworks explained 3.0% of genetic heritability (SE = 0.79%), while the brain subnetworks explained 7.4% (SE = 1.0%), highlighting a stronger importance of the brain network. These are substantially higher than what was explained by the top 93 significant SNPs at three loci reported in the original GWAS, which only explained 1.1% (SE = 0.0039) of heritability. Notably, the total SNP heritability estimated by all GWAS SNPs is 11.8% [10], and our brain and peripheral networks together explained a majority of this (10.4%).

## Discussion

ASD is a neurodevelopmental disorder that possesses complex heterogeneity in its genetic architecture [20]. Previous research has uncovered both common and rare variants in the pathogenesis of ASD, and have implicated the central nervous system as well as various peripheral tissues and biological pathways that interact to affect ASD [5, 7, 12–19, 85, 86]. However, a leading challenge in our understanding of ASD is whether the common and rare variants inform on similar or different mechanisms that can be targeted for therapy. If the former, generalized therapeutic approaches may be developed to target ASD; if the latter, precision approaches are needed to target individual subtypes of ASD.

In our study, we utilize summary statistics from an ASD GWAS [10], tissue-specific eQTL/sQTL data from the GTEx database [41], and tissue-specific gene coexpression and regulatory networks to investigate ASD. Considering both common and rare variants, we sought to understand the molecular interactions in a broad range of brain and peripheral tissues to uncover tissues, genes, biological pathways, and gene networks that are enriched for either common or rare variants of ASD in a completely data-driven manner without using prior knowledge as input. Furthermore, we aimed to predict key drivers within gene regulatory networks that may be of interest for further mechanistic and experimental studies.

From our marker set enrichment analysis (MSEA) of the ASD GWAS, we found a diverse range of tissues and gene coexpression modules enriched for ASD common variants. While top enriched modules are mainly from brain tissues such as the anterior cingulate cortex and the amygdala, there were also peripheral tissues whose modules showed relevance to ASD GWAS signals (Supplementary Table 3). The coexpression module enrichments of the anterior cingulate cortex and amygdala are consistent with previous findings that observed structural abnormalities, dysregulations and alterations in both ASD mouse models and adult ASD individuals [87–89]. Other brain regions (frontal cortex [BA9], cerebellum, broader cortical tissue) which had an enrichment of modules for ASD, were previously found to exhibit abnormal development in the disorder [90–92]. Thus, our findings on these brain regions harboring ASD common variants provide additional evidence supporting their importance in cognitive function, motor coordination, and neuronal activity in the context of ASD.

In addition to these confirmatory outcomes of various brain regions, we found that tissues in the digestive, reproductive, endocrine, and immune systems also displayed ASD GWAS enrichment within particular coexpression modules. The digestive system has recently become heavily implicated in ASD pathogenesis as research has continued to explore the interactions within the microbiota-gut-brain axis, and how there are distinctive gastrointestinal complications present in those with ASD [19, 93]. Our findings on the reproductive system support research on how maternal factors and their influence on fetal development contribute to ASD risk [94–99], which can occur via hormones or immune pathways. Our genetics-driven findings on this multi-system involvement highlight the complexity of the genetic architecture across both brain and peripheral regions of the body in the pathogenesis of ASD.

Our exploration of pathway annotations from these coexpression modules enriched for ASD GWAS signals further emphasize how the complexity of ASD spans across diverse biological pathways. We found that immune, cellular signaling, and cell growth and regulation pathways were represented among significant modules from various tissues (Figs. 2, 3). Pathways related to Alzheimer’s disease and Amyotrophic Lateral Sclerosis were consistently enriched among modules from various brain regions, which interestingly aligns with their neurological dysfunction and potential associations with ASD [100–104].

We also noticed that tissues and pathways involved in the endocrine and immune systems were highly represented among the ASD-associated modules. The male and female reproductive, digestive, and adipose systems contained various pathways involved in immune regulation (e.g., Interleukin-6, CD40 pathways), metabolic functions (e.g., cysteine and methionine metabolism, fatty acid synthesis, bile acid metabolism), structural integrity and development (hyaluronan uptake and degradation), or cellular signaling, processes, and regulation (e.g., MAPK pathways, EDG1 pathways, ENOS activation). mTOR signaling, critical for the regulation of metabolism, immune function, and cellular homeostasis [57], is also among the consistent pathways across systems. Our results support the role of immune dysregulation and metabolic dysfunction in ASD pathogenesis [105–109] and implicates the interconnected nature between pathways across systems and their collective contribution to the diversity and complexity of ASD pathogenesis. As a whole, our MSEA findings recapitulate previous studies that highlight the impact of altered neural functioning in various brain regions, and also provide causal inference through genetic variants supporting the roles that digestive, reproductive, endocrine, and immune system have in ASD development.

In our key driver analysis, we discovered that the brain outperforms peripheral tissues in terms of both the abundance of significant rare and common variants among the key drivers identified and the enrichment in their subnetworks for genes with either rare or common variants (Fig. 4; Fig. 5A, B). As expected, a greater number of brain tissue key driver subnetworks contained a significant number of both rare and common variants, which suggest that these key drivers are crucial regulators within ASD pathogenesis given the convergence of these two types of variants (Fig. 5C). The rankings of key driver subnetworks in terms of their rare or common variant enrichment highlight the central role of the brain in ASD, yet also point towards the digestive system as the leading peripheral region for common variant abundance (Fig. 5A, B).

Our analysis also highlighted top brain key driver genes such as *SYT1* (Fig. 6A) and *ADD2* (Fig. 6B). Our detailed exploration of these key driver subnetworks strongly support their potential role in orchestrating genes with either rare or common ASD variants to perform key neuronal functions. While *SYT1* has previously been found to be a gene with known rare variant of ASD [51], and a common variant of *ADD2* has been implicated in the disorder as well [10], our network analysis highlights the unexplored tissue-specific network interactions between these key drivers and other ASD genes with key functions in neuronal excitability, homeostasis, and neurotransmitter release (e.g., *SCN8A, AMPH, ATP9A*, shown in Supplementary fig. 1A-C) carrying both types of variants. By contrast, *PRR36* (Supplementary fig. 2A), a gene associated with other ASD-related genes such as *SHANK3* (implicated in synaptic organization and neurodevelopment [82]) highlights potential ASD mechanisms driven by common variants in its network. Furthermore, the identification of shared KDs between the brain and digestive tissues as seen in *PCDH7* (Supplementary fig. 2B), implicates converging mechanisms across tissues that may individually or collectively influence ASD. Overall, these networks provide the opportunity for future experimentation and validation to test how multiple ASD genes interact to affect ASD pathogenesis.

Altogether, our systems genetics approach to studying both ASD common and rare variants across the brain and periphery not only confirmed numerous previous findings but revealed new avenues for future exploration. Each of our top 10 coexpression modules (Supplementary Table 3) captured tissue origins and pathways with known relevance in ASD pathogenesis, with cellular processes and immune pathways being the most replicated across brain and peripheral tissues (Figs. 2C, 3). The abundance of both brain and peripheral coexpression modules enriched for ASD GWAS signals (Fig. 2B) supports the growing body of research that suggests ASD relevance within not only the brain but the digestive, endocrine, and immune systems [18, 19, 93, 96, 99, 110], and further provides potential causal inference for their involvement due to the incorporation of genetic variants in our analysis. The notable convergence of known rare and common variants in the brain and the identified key drivers regulating ASD genes offer prioritized targets for future mechanistic and therapeutic studies.

Our partitioned heritability analysis revealed that brain subnetworks account for a substantially larger share of ASD heritability (7.0%) than peripheral subnetworks (3.4%), which is consistent with ASD’s core neurodevelopmental basis. However, the finding that peripheral subnetworks contribute ~3% of liability variance provides genetic support for systemic involvement, including immune and metabolic pathways, in ASD pathogenesis. Both explain substantially higher heritability than top genome-wide significant hits (1%), highlighting the value of integrative, network-based approaches for uncovering hidden components of genetic architecture. These results are analogous to findings in other diseases, where tissue-specific networks captured disease-relevant heritability far beyond genome-wide significant hits [26, 35].

### Limitations of the study

We note the following limitations in our study. First, while we attempt to use the largest ASD GWAS along with functional genomics datasets such as GTEx with the most comprehensive coverage of tissue-specific gene regulation, the demographics of both the GWAS and the GTEx datasets is primarily of central European origin. ASD research is constantly updating and new discoveries continue to be made, so future studies with expanded population coverage are warranted when such data becomes available. Resources that are actively updated such as the Simons Foundation for Autism Research Initiative [51], the Australia Autism Biobank [111], and the MSSNG database [112] will undoubtedly provide additional useful data for future studies. Secondly, our studies mainly integrated genetic and transcriptomic data to model genetically perturbed gene networks in ASD. Further incorporation of transcriptome, metabolome, and epigenome datasets of ASD [113–115] is necessary to expand our understanding. Third, we recognize that we did not apply our analyses directly to rare variants; instead, we used genes informed by rare variants curated from the SFARI database across multiple studies. Future work could extend our Mergeomics framework to individual rare variant datasets to reveal additional insights. We also acknowledge that we have only performed a tissue-level analysis. Cell-level analysis is a fast-growing area of research across numerous fields, and ASD is a disease that demands this level of cellular specificity. Lastly, there is a male bias in the ASD prevalence [110], which emphasizes that a sex-specific analysis is likely to reveal additional key insights. Expanding data coverage and performing cell-level, sex-specific analyses are pivotal directions for future research to provide a higher-resolution understanding of ASD’s genetic complexities. Overall, our findings will assist in the generation of new hypotheses and experimental validations to uncover new mechanisms and guide therapeutic development for neurodevelopmental disorders such as ASD.

## Supplementary information

Supplementary Figure 1

Supplementary Figure 2

Supplementary Materials

Supplementary Table 4

Supplementary Table 5

## Data Availability

The R package for Mergeomics can be found at https://bioconductor.org/packages/release/bioc/html/Mergeomics.html.
