# Supplementary material for: Convergence and divergence of genes informed by common and rare variants of autism spectrum disorders in tissue-specific pathways and gene networks
Source: Transl Psychiatry. 2026 Feb 6;16:98. doi: 10.1038/s41398-026-03824-x (PMC12923589; doi:10.1038/s41398-026-03824-x)
Supplement: Supplementary file 3 — Supplementary Materials [file 41398_2026_3824_MOESM3_ESM.docx]

**SUPPLEMENTARY FIGURE LEGENDS**

**Supplementary Figure 1: Brain tissue key driver subnetworks demonstrating convergence of rare and common variants: SCN8A, AMPH, ATP9A.**

Key driver subnetworks **A)** SCN8A (sodium voltage-gated channel alpha subunit 8), **B)** AMPH (amphiphysin), and **C)** ATP9A (ATPase phospholipid transporting 9A) were identified within the brain Bayesian network and showed high enrichment of both rare and common ASD-associated variants. These key drivers and their first-degree network neighbors were visualized using Cytoscape and colored based on their SFARI database ASD confidence level stratification or by their ASD GWAS common variant disease association strength. Uncolored nodes were genes within the Bayesian network but were not found to be associated with either grouping. EnrichR was used to analyze the key driver subnetwork genes to generate pathway annotation terms, with the top annotations displayed below each network. Terms were ranked based on their *-log10* p-value.

**Supplementary Figure 2: Peripheral tissue key driver subnetworks demonstrating high enrichment of common variants or a convergence of rare and common variants: PRR36 and PCHD7.**

Key driver subnetworks **A)** PRR36 (proline rich 36), and **B)** PCDH7 (protocadherin 7) were identified from the endocrine and digestive Bayesian networks, respectively. PRR36 displayed high enrichment of common variants while PCDH7 showed a convergence of both rare and common variants. These key drivers and their first-degree network neighbors were visualized using Cytoscape and colored based on their SFARI database ASD confidence level stratification or by their ASD GWAS common variant disease association strength. Uncolored nodes were genes within the Bayesian network but were not found to be associated with either grouping. EnrichR was used to analyze the key driver subnetwork genes to generate pathway annotation terms, with the top annotations displayed below each network. Terms were ranked based on their *-log10* p-value.

# **SUPPLEMENTARY TABLES**

## **Supplementary Table 1. Summary of eQTLs and sQTLs from the Genotype Tissue Expression Portal (GTEx) database for Mergeomics analysis.**

eQTLs and sQTLs from 14 brain regions and 35 peripheral tissues were retrieved from the GTEx database to be used for tissue-specific mapping of SNPs from the ASD GWAS to genes that are potentially regulated by the SNPs in each tissue. Four tissues did not have eQTL/sQTL data (bladder, endocervix, fallopian tube, renal medulla), so distance-based mapping was utilized for their respective tissues during Marker Set Enrichment Analysis.

| **Region** | **Tissue** | **eQTL Count** | **sQTL Count** |
| --- | --- | --- | --- |
| Peripheral | Adipose (Subcutaneous) | 1501725 | 649559 |
| Peripheral | Adipose (Visceral Omentum) | 1116992 | 501342 |
| Peripheral | Adrenal Gland | 606549 | 266580 |
| Brain | Amygdala | 241511 | 84934 |
| Brain | Anterior Cingulate Cortex (Brodmann Area 24) | 360811 | 121320 |
| Peripheral | Artery (Aorta) | 1137522 | 446515 |
| Peripheral | Artery (Coronary) | 468104 | 231587 |
| Peripheral | Artery (Tibial) | 1528215 | 615729 |
| Peripheral | Breast Mammary Tissue | 915137 | 478320 |
| Brain | Caudate | 592299 | 183573 |
| Brain | Cerebellar Hemisphere | 689639 | 234153 |
| Brain | Cerebellum | 838323 | 276313 |
| Peripheral | Colon (Sigmoid) | 861273 | 367653 |
| Peripheral | Colon (Transverse) | 946365 | 392568 |
| Brain | Cortex | 675220 | 208046 |
| Peripheral | Cultured Fibroblasts | 1558412 | 604866 |
| Peripheral | EBV-transformed Lymphocytes | 303559 | 215630 |
| Peripheral | Esophagus (Gastroesophageal Junction) | 891705 | 375284 |
| Peripheral | Esophagus (Mucosa) | 1374993 | 480793 |
| Peripheral | Esophagus (Muscularis) | 1344979 | 499704 |
| Brain | Frontal Cortex (Brodmann Area 9) | 511541 | 168139 |
| Peripheral | Heart (Atrial Appendage) | 962118 | 360824 |
| Peripheral | Heart (Left Ventricle) | 850330 | 286330 |
| Brain | Hippocampus | 374601 | 128103 |
| Brain | Hypothalamus | 385593 | 147721 |
| Peripheral | Kidney Cortex | 76350 | 35421 |
| Peripheral | Liver | 407126 | 158972 |
| Peripheral | Lung | 1261501 | 567609 |
| Peripheral | Minor Salivary Gland | 294404 | 143965 |
| Peripheral | Muscle (Skeletal) | 1405527 | 574674 |
| Peripheral | Nerve (Tibial) | 1720751 | 659871 |
| Brain | Nucleus Accumbens | 580998 | 199042 |
| Peripheral | Ovary | 370124 | 197505 |
| Peripheral | Pancreas | 818024 | 251970 |
| Brain | Pituitary | 713991 | 326113 |
| Peripheral | Prostate | 514066 | 258156 |
| Brain | Putamen | 477659 | 137298 |
| Peripheral | Skin (Non-Sun-Exposed Suprapubic) | 1409730 | 564897 |
| Peripheral | Skin (Sun-Exposed Lower Leg) | 1632271 | 638497 |
| Peripheral | Small Intestine (Terminal Ileum) | 414021 | 195522 |
| Brain | Spinal Cord (Cervical C1) | 293087 | 111631 |
| Peripheral | Spleen | 770698 | 279622 |
| Peripheral | Stomach | 706670 | 295700 |
| Brain | Substantia Nigra | 198962 | 75770 |
| Peripheral | Testis | 1554886 | 954055 |
| Peripheral | Thyroid | 1765762 | 682833 |
| Peripheral | Uterus | 216551 | 138022 |
| Peripheral | Vagina | 224692 | 139644 |
| Peripheral | Whole Blood | 1276546 | 377472 |

## **Supplementary** **Table 2: Summary of tissue-specific Bayesian networks constructed using GTEx transcriptome data.**

Bayesian networks were constructed for individual tissues and further merged into seven Bayesian networks representing major biological systems to reduce sparsity.

| **Network Name** | **Individual Tissues** | **Node Number** | **Edge Number** |
| --- | --- | --- | --- |
| Adipose | Adipose (Subcutaneous), Adipose (Visceral Omentum) | 2262 | 17758 |
| Brain | Amygdala, Anterior Cingulate Cortex, Caudate, Cerebellar Hemisphere, Cortex, Frontal Cortex, Hippocampus, Hypothalamus, Nucleus Accumbens, Putamen, Pituitary, Spinal Cord, Substantia Nigra | 37866 | 223949 |
| Cardiovascular | Artery (Aorta), Artery (Coronary), Artery (Tibial), Heart (Atrial Appendage), Heart (Left Ventricle) | 11241 | 41168 |
| Digestive | Colon (Sigmoid), Colon (Transverse), Esophagus (Gastroesophageal Junction), Esophagus (Mucosa), Esophagus (Muscularis), Liver, Minor Salivary Gland, Pancreas, Small Intestine (Terminal Ileum), Stomach | 14259 | 68401 |
| Endocrine | Adrenal, Ovary, Pituitary, Testis, Thyroid | 14720 | 37690 |
| Female Reproductive | Breast Mammary Tissue, Endocervix, Ectocervix, Fallopian Tube, Ovary, Uterus, Vagina | 10020 | 25117 |
| Immune | EBV Lymphocytes, Spleen, Whole Blood | 10430 | 21815 |
| Immune-Adipose | Adipose (Subcutaneous), Adipose (Visceral Omentum), EBV Lymphocytes, Spleen, Whole Blood | 12692 | 39573 |

**Supplementary Table 3: Top 10 coexpression modules enriched for ASD GWAS signals from Marker Set Enrichment Analysis (MSEA).**

The top ten gene sets from MSEA were ranked based on false discovery rate (FDR) of ASD GWAS enrichment. Tissue corresponds to the tissue origin of the gene coexpression modules from GTEx data; module name indicates which coexpression module within a given tissue showed ASD GWAS enrichment. The top annotations indicate the pathway terms that had the highest enrichment p-values based on pathway annotation of the genes in each tissue-specific coexpression module. The top informative genes were selected based on the SNP p-values from the ASD GWAS, prioritizing those with previous evidence of association with ASD, including known coding genes and others with established roles in ASD pathogenesis or pathophysiology.

|  | **Tissue** | **Module** | **FDR** | **Top Annotations** | **Top Informative Genes in GWAS** |
| --- | --- | --- | --- | --- | --- |
| **1** | Anterior Cingulate Cortex | blue | 1.27E-24 | Protein Degradation, Long Term Potentiation, Immune System | *AF131216.5, ARL17A, CTC-498M16.4, GABBR1, KANSL1, LRRC37A, MAPK8IP1P2, MAPT, PLEKHM1, TMEM161B-AS1* |
| **2** | Amygdala | black | 3.07E-24 | Spliceosome Activity, Proteasome Activity, Electron Transport | *ARL17A, CRHR1, GABBR1, KANSL1, LINC02210, LRRC37A, LRRC37A2, MAPK8IP1P2, MAPT, PLEKHM1* |
| **3** | Liver | turquoise | 5.59E-23 | Cell Cycle Regulation, DNA Repair, mTORC1 Activity | *ARL17A, DND1P1, KANSL1, KANSL1-AS1, LINC02210, LRRC37A2, MAPK8IP1P2, MAPT-IT1, PLEKHM1, RP11-259G18.3* |
| **4** | EBV Lymphocytes | blue | 2.01E-19 | TNF-α Signaling, Pancreatic Cancer, Neurotrophin Signaling | *ARHGAP27, BLK, DND1P1, FAM167A, HLA-A, KANSL1, LINC02210, MAPK8IP1P2, RP11-259G18.3, RP11-707O23.1* |
| **5** | Minor Salivary Gland | light-green | 6.69E-19 | RNA Splicing, TGF-β signaling, Membrane Trafficking | *ARL17A, FAM85B, KANSL1, KANSL1-AS1, LINC02210, LRRC37A2, LRRC37A4P, MAPK8IP1P2, RP11-259G18.1, RP11-259G18.3* |
| **6** | Frontal Cortex | gray60 | 7.77E-19 | Immune Response and Regulation, Neuronal, Synaptic Processes | *ARL17A, CRHR1, GABBR1, KANSL1, KIZ, LINC02210, LRRC37A, MAPT, PLEKHM1, TDH* |
| **7** | Uterus | turquoise | 3.33E-16 | Transcription, mRNA processing | *ARL17A, DND1P1, KANSL1, KANSL1-AS1, LINC02210, LRRC37A, LRRC37A2, MAPK8IP1P1, RP11-259G18.3, RP11-707O23.1* |
| **8** | Cerebellum | purple | 1.04E-15 | Energy Metabolism, Cellular Stress Response, Oxygen Transport | *ARL17A, CRHR1, FMNL1, GABBR1, KANSL1, LINC02210, MAPT, NSF, PLEKHM1, TDH* |
| **9** | Cortex | blue | 2.49E-15 | Olfactory Signal Transduction | *ARHGAP27, CRHR1, DND1P1, GABBR1, KANSL1, LINC02210, LRRC37A, MAPT, PLEKHM1, RP11-259G18.1* |
| **10** | Testis | magenta | 2.52E-13 | Cell Cycle Regulation, DNA Repair | *LINC00208, MACROD2, MSRA, PINX1, PTBP2, RP11-177H2.2, RP11-227D2.3, RP11-6N13.1, TDH, XRN2* |

## **Supplementary Table 4: Complete list of ASD Enriched module from MSEA analysis.**

Uploaded as a separate Excel file.

**Supplementary Table 5: Comparison of tissue-specific modules identified in MSEA and tissue-matched differentially expressed genes (DEGs) from previous transcriptomic studies.**

Uploaded as a separate Excel file.

**Supplementary Table 6: Enrichment of brain key drivers across SFARI levels.**

| **SFARI Level** | **Enrichment p-Value** | **Fold Change** |
| --- | --- | --- |
| **1** | **8.35e-36** | **4.26** |
| **2** | **5.84e-59** | **3.30** |
| **3** | **2.59e-14** | **4.05** |
| **S** | **3.08e-33** | **3.81** |

## **Supplementary Table 7: Top network key drivers in brain and peripheral tissues with subnetwork enrichment for both rare variants (RV) and common variants (CV) of ASD at an enrichment p-value < 0.05.**

| **KD** | **RV Score** | **CV Score** | **Tissue** | **Tissue Region** |
| --- | --- | --- | --- | --- |
| **ADD2** | 2.07E-09 | 8.12E-07 | Substantia Nigra, Pituitary, Frontal Cortex | Brain |
| **AMPH** | 1.43E-04 | 4.63E-03 | Substantia Nigra | Brain |
| **ATP9A** | 8.83E-06 | 6.23E-04 | Cerebellum | Brain |
| **CALD1** | 2.22E-03 | 2.49E-02 | Colon Sigmoid | Digestive |
| **CD74** | 3.31E-02 | 5.32E-04 | Colon Sigmoid | Digestive |
| **DYNC1H1** | 1.17E-02 | 7.85E-03 | Cerebellum | Brain |
| **DYNC1I1** | 3.01E-02 | 1.17E-02 | Cerebellum | Brain |
| **MAP2K1** | 1.91E-04 | 3.12E-02 | Caudate | Brain |
| **PCDH7** | 1.61E-05 | 3.66E-02 | Colon Sigmoid | Digestive |
| **PLPP3** | 6E-04 | 3.69E-02 | Putamen, Frontal Cortex | Brain |
| **RBFOX2** | 2.42E-05 | 1.39E-02 | Substantia Nigra | Brain |
| **SCN8A** | 5.99E-04 | 6.5E-04 | Substantia Nigra | Brain |
| **SNAP91** | 9.16E-08 | 1.68E-02 | Substantia Nigra | Brain |
| **SYP** | 7.96E-04 | 2.18E-02 | Substantia Nigra, Pituitary | Brain |
| **TMEM130** | 3.45E-08 | 1.24E-03 | Pituitary | Brain |
| **XKR4** | 2.13E-02 | 6.79E-03 | Colon Sigmoid | Digestive |
